# One Year Assessment of the Hearing Preservation Potential of the EVO Electrode Array

**DOI:** 10.3390/jcm10235604

**Published:** 2021-11-29

**Authors:** Nicolas Guevara, Cécile Parietti-Winkler, Benoit Godey, Valerie Franco-Vidal, Dan Gnansia, Marine Ardoint, Michel Hoen, Chadlia Karoui, Eric Truy, Christophe Vincent, Isabelle Mosnier, Yann Nguyen

**Affiliations:** 1 Institut Universitaire de la Face et du Cou (University Institute of the Face and Neck), Centre Hospitalier Universitaire de Nice, Université Côte d’Azur, 06000 Nice, France; guevara.n@chu-nice.fr; 2 Service d’ORL et CCF, CHU Nancy, 54000 Nancy, France; c.parietti@chru-nancy.fr; 3 Laboratoire Développement, Adaptation et Handicap (DevAH-EA 3450), Faculty of Medicine and Faculty of Sciences and Sport, University of Lorraine, Vandoeuvre-lès-Nancy, 54000 Nancy, France; 4 Department of Otolaryngology, Head Neck Surgery, University Hospital Ponchaillou, 35000 Rennes, France; benoit.godey@chu-rennes.fr; 5 Ear, Nose, and Throat Department, Bordeaux University Hospital, 33000 Bordeaux, France; valerie.vidal@chu-bordeaux.fr; 6 Research & Technology, Oticon Medical, Technologies and Gene Therapy for Deafness, Hearing Institute, Institut Pasteur/Inserm, 16096 Paris, France; dagn@oticonmedical.com (D.G.); mard@oticonmedical.com (M.A.); mhoe@oticonmedical.com (M.H.); 7 Service d’ORL, De Chirurgie Cervico-Faciale et D’audiophonologie, Hôpital Edouard Herriot, Hospices Civils de Lyon, Université de Lyon, 69000 Lyon, France; eric.truy@chu-lyon.fr; 8 Centre de Recherches en Neurosciences de Lyon, Équipe IMPACT, INSERM UMRS1028 CNRS UMR5292, 69000 Lyon, France; 9 Otology and Oto-Neurology Department, Lille University Hospital, 59000 Lille, France; christophe.vincent@chru-lille.fr; 10 ENT Department, Pitié Salpêtrière Hospital, APHP/Sorbonne Université, 75013 Paris, France; isabelle.mosnier@aphp.fr (I.M.); yann.nguyen@inserm.fr (Y.N.); 11 Institut de L’audition Pasteur/Inserm UMR 1120, 75012 Paris, France

**Keywords:** cochlear implantation, hearing preservation, HEARRING classification, functional hearing, EVO electrode array

## Abstract

Background: A prospective longitudinal multicentre study was conducted to assess the one-year postsurgical hearing preservation profile of the EVO^TM^ electrode array. Methods: Fifteen adults presenting indications of electro-acoustic stimulation (pure-tone audiometry (PTA) thresholds ≤70 dB below 750 Hz) were implanted with the EVO™ electrode array. Hearing thresholds were collected at five time-points from CI activation to twelve months (12M) after activation. Hearing thresholds and hearing preservation profiles (HEARRING group classification) were assessed. Results: All subjects had measurable hearing thresholds at follow-up. No case of complete loss of hearing or minimal hearing preservation was reported at any time point. At activation (N_act_ = 15), five participants had complete hearing preservation, and ten participants had partial hearing preservation. At the 12M time point (N_12m_ = 6), three participants had complete hearing preservation, and three participants had partial hearing preservation. Mean hearing loss at activation was 11 dB for full range PTA and 25 dB for PTAs low-frequency (125–500 Hz). Conclusions: This study provides the first longitudinal follow-up on associated hearing profiles to the EVO™ electrode array, which are comparable to the literature. However, other studies on larger populations should be performed.

## 1. Introduction

The cochlear implant (CI) can constitute a dilemma for subjects with severe high-frequency hearing loss (HL). On the one hand, this population mostly does not benefit from conventional hearing aids. On the other hand, a traumatic CI insertion could damage their low-frequency residual hearing. Preservation of the cochlear structure is key to the success of cochlear implantation for this population. When these subjects are CI candidates (averaged hearing thresholds <80 dB HL according to recommendations of CI manufacturers), they may be offered electro-acoustic stimulation (EAS). However, EAS is only possible if the candidate has sufficient functional residual hearing after surgery. With the increasing understanding of the mechanisms underlying intracochlear trauma and the establishment of predictive factors associated with low-frequency hearing preservation (HP), recent decades have witnessed an evolution towards atraumatic surgery [1,2], accompanied by an evolution of CI components [3]. Atraumatic surgery has been shown to improve HP scores [4,5]. Implant manufacturers have improved their electrode arrays by making them thinner and more flexible [6], aiming for trauma-free insertion so that the cochlear structures can be preserved. The length of the electrode array is also a key variable. For example, Gantz et al. [7] worked on a short 10 mm array, the aim being not to damage the apical, low-frequency encoding cells. They were able to preserve residual hearing partially or completely in 96% of cases. Residual hearing can last years after implantation [8,9], even in elderly patients [10], but it is also often lost; in the latter case, electrical stimulation from the CI would be the only route to provide hearing, and a 10 mm array would not be as efficient as a standard-length array, because of its reduced cochlear coverage. A reimplantation would then be more appropriate [11]. It is therefore recommended to insert over at least 360°, which corresponds to 18–24 mm (variable depending on the anatomy of the cochlea) of insertion, for a frequency region close to 1 kHz [12,13]. This appears to be a good compromise to preserve residual hearing and obtain performance with the CI alone if residual hearing post-implantation is lost [14]. 

The EVO^TM^ electrode array has been designed to preserve hearing. This 24 mm long electrode array carries 20 electrodes. Its narrow diameter and flexibility, as well as its soft surface and round tip, were designed for lower-trauma insertion [15]. Extra-cochlear rings have been placed to facilitate insertion and decrease insertion force. A previous study with the EVO^TM^ electrode array showed that the insertion forces used during implantation were considerably reduced—over 32% compared to the standard electrode array [16]. Bento et al. [17] evaluated the combination of a soft-surgery procedure implicating round-window insertion, the use of dexamethasone and hyaluronic acid during surgery, with the use of the EVOTM electrode array on hearing preservation in seven participants with low-frequency functional hearing. Their preliminary outcomes described hearing threshold shifts after activation of 24.5 ± 18.2 dB HL up to 500 Hz and 21.8 ± 19.2 dB up to 1 kHz. To date, no longitudinal study has investigated the atraumatic and HP profile of CI users with the EVO^TM^ electrode array only. 

The purpose of the present study was to evaluate the atraumatic nature of the EVO^TM^ electrode array with the assessment of HP and functional hearing profiles up to twelve months after CI activation.

## 2. Materials and Methods

This prospective multicentric clinical investigation was carried out in accordance with the regulations in force based on the provisions stipulated in the Declaration of Helsinki and was registered in the U.S. National Library of Medicine ClinicalTrials.gov database (identifier NCT02966379). For this purpose, the favourable opinion of the CPP was required and obtained (RCB:2015-A00445-44), as well as the authorization of the French health authorities (ANSM). Clinical research was carried out in accordance with good clinical practices (ICH standard) and NF ISO 14155:2011 and in accordance with the MR001 reference methodology of the French National Information Science and Liberties Commission (CNIL). Participants were included in seven French CI centres (Lille, Bordeaux, Paris Pitié Salpêtrière, Nice, Nancy, Lyon, Rennes). The investigation occurred between April 2016 and April 2019. 

### 2.1. Participant Selection

To be included, CI candidates had to be adults (≥18 years old) with post-lingual deafness onset, be fluent in French and follow the standard post-implantation follow-up and rehabilitation program. All procedures were primary cochlear implantations. Tonal audiometry had to be showing functional auditory residual hearing in low frequencies (pure-tone audiometry (PTA) thresholds ≤70 dB up to 500 Hz included) prior to implantation. Tonal threshold at non-aided condition had to be measurable on at least one frequency before activation of the implant. Written informed consent was obtained from each patient to participate in the study and to receive the Digisonic^®^ SP cochlear implant, including the EVO^TM^ electrode array and an electro-acoustic prototype sound processor (EAS)—(Oticon Medical, Vallauris, France). Fifteen adult CI candidates were initially included in the study. All their demographics are summarized in Table 1. 

The study included eight time points: two preliminary visits, the surgery and five follow-up visits. The two pre-operative visits consisted of the inclusion visit and the auditory preoperative assessment visit in a two-month period prior to the CI surgery. Unaided auditory hearing levels were measured using free-field warble tone thresholds at 125, 250, 500, 750, 1000, 2000 and 4000 Hz. The EAS activation occurred one month after cochlear implantation. At time points after activation, if at least one hearing threshold was detectable, all hearing thresholds were measured. If not, the participant was equipped with a Saphyr^®^ sound processor providing electric-only stimulation (ES)—(Oticon Medical, Vallauris, France) and exited from the protocol. This was repeated at one (1M), three (3M), six (6M) and twelve months (12M) after activation. At 6M and12M time points, participants were also given the choice to keep the EAS or not.

### 2.2. Surgical Procedure

To assess the success of atraumaticity of the EVOTM electrode array, a standard CI surgery procedure based on round window insertion was preferred. Recommendations were that the surgeon accesses the cochlea by posterior tympanotomy, locates the round window and goes through to insert the electrode array. The implant body had to be fixed before opening the round window. The opening time of the round window had to be less than 2 min. The surgeon inserted the electrode array into the tympanic ramp following the cochlear spiral and finalized the insertion by resting on the push rings of the array. In case of difficult insertion, the surgeon was advised to stop at the first point of resistance. Corticosteroids were systematically administered to the participants (1 mg/kg solumedrol). 

### 2.3. Outcomes

The first outcome measure was the change in hearing thresholds at 125, 250, 500, 750, 1000, 2000 and 4000 Hz, assessed between pre- and post-implantation at activation and 1M, 3M, 6M and 12M time points. The second outcome measure consisted of the assessment of residual HP after implantation of the EVO^TM^ electrode array. The HEARRING Group approach ([18]—https://www.hearring.com/ accessed on 12 October 2021) was used to classify the different HP profiles in the study population (Table S1). The HEARRING formula expresses post-operative thresholds as a percentage of pre-operative threshold and corrects by the upper limit of measurable thresholds as described in Formula (1):

HP = [1 − (((PTApost − PTApre))/((PTAmax − PTApre))) ×100] (%)(1)

where PTA post is the pure tone average threshold measured postoperatively, PTA pre is pure tone average measured pre-operatively, PTA max is the compliance limits of the audiometer and HP is the hearing preservation numerical scale in percent [18]. The maximal hearing level of the audiometer was the same as that defined by the HEARRING group for each frequency: 125 Hz (90 dB), 250 Hz (105 dB), 500 Hz (110 dB) and 750 Hz (115 dB). Depending on the HP scale, the HP could be complete (if >75%), partial (if ranging between 25 and 75%) or minimal (if <25%), as suggested by The HEARRING classification. If no measurable hearing is possible, it is classified as complete loss of hearing. To assess the change in hearing threshold over the different time points after activation, mean hearing loss (HL) as PTA difference between one time point and a preceding time point was calculated both at full range audiometry (125–4000 Hz) and in the low frequency region (125–500 Hz). Functional hearing at low-frequency regions was also investigated and defined as low-frequency PTA (0.125–0.5 kHz) ≤ 80 dB (HL), as in recent studies [19,20]. To further explore the data regarding subjects with functional hearing, low-frequency HP was also assessed using the standard classification [9,21], defined as complete preservation if mean hearing threshold shift ≤10 dB; partial preservation for a mean shift between 10 dB and 30 dB; and minimal preservation if shift ≥30 dB. Loss of residual hearing would be defined when no measurable threshold is possible at maximum hearing levels. 

### 2.4. Statistical Analyses

All quantitative data were presented as mean, standard deviation and median. Analyses were conducted using RStudio Team (RStudio Inc., Boston, MA, USA). For each analysis on hearing threshold follow-up, a linear mixed-effect (LME) model for repeated measures was fitted to the data with a random intercept for participants and a fixed effect for frequency and month as covariates. To further explore the collected data, a repeated-measures ANOVA and post hoc pairwise comparisons were conducted. Statistical significance was defined as *p* ≤ 0.05. Some demographic data were missing when performing the analysis (i.e., n.a. in Table 1). These data were not included in any analysis. Correlation analysis was performed using a Pearson test.

## 3. Results

Fifteen CI users (mean age = 60.7 ± 17 years, range (31:86), 6 males and 9 females) underwent CI surgery with a unilateral EVO™ electrode array, using the HP surgical procedure (5 right-sided CIs, 10 left-sided CIs). Full insertion of the electrode array was achieved in 53.3% of the cases, in agreement with the instruction given to surgeons to stop insertion at first resistance to prioritize atraumaticity of the insertion over full insertion. Mean number of inserted electrodes was 18.8 ± 1.4, range (17:20). At activation time point, mean number of activated electrodes was 18.5 ± 1.6, range (16:20). Hearing thresholds were collected for all participants up to 3M but for one participant who decided to step-out of the study for personal reasons (non-adherence to protocol constraints) (i.e., sample size at activation time point: N_act_ = 15, sample size at the 1M time point: N1m = 15, sample size at the 3M time point: N_3m_ = 14). At the 6M time point, as participants were given the choice of changing the sound processor, and one participant decided to be fitted with the ES and thus left the study (i.e., sample size at the 6M time point N_6m_ = 13). At the 12M time point, six participants chose to remain with the EAS (i.e., sample size at the 12M time point: N_12m_ = 6), while seven participants quit the study due to personal constraints or preference for an ES trial. All collected individual PTAs are presented in Table 2. HP score percentages and classification according to the HEARRING group along with follow-up time points are available in Supplemental Material 1 both for the full range PTAs and PTAs of low-frequency (0.125–500 Hz). 

### 3.1. Early Follow-Up Data

**Figure 1:**
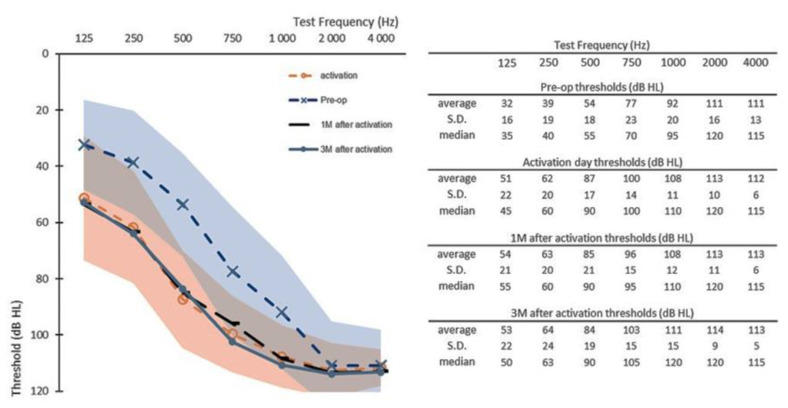
Pre-operative hearing thresholds (Pre-op, *n* = 15), post-operative hearing thresholds at activation day (activation, *n* = 15), at one month after activation (1M, *n* = 15) and at three months after activation. (3M, *n* = 14). Shaded areas represent the maximal and minimal standard deviations of the pre-operative data (in blue) and of the activation day data (in orange). S.D. = standard deviation.

At activation (Nact = 15), all participants had measurable hearing thresholds, as described in Figure 1. Linear mixed-effect analysis revealed that changes in hearing thresholds between time points were not all similar, with at least one change in the three time points as statistically significantly different from others (F (3, 371)= 34.8, *p* < 0.001) and were as well not all similar with at least one change at one of the five test-frequencies as statistically significantly different from the others (F (6, 370) =194.4, *p* < 0.001) with a significant interaction effect between frequency and time point (F (18, 371) = 2.4, *p* = 0.001). Post hoc pairwise multi-comparisons indicated that changes at activation, at 1M and at 3M, were significantly higher from hearing thresholds measured at the preoperative visit between 125 Hz and 1000 Hz. No significant differences were found at both 2000 Hz and 4000 Hz during follow-up. No significant difference was found between post-operative hearing thresholds across subjects. 

Over the early follow-up period, no participant showed a total loss of hearing or a minimal HP according the HEARRING classification. At activation (N_act_ = 15), five participants (33.3%) had complete HP (range = (84:102)%), and ten participants (66.7%) had partial HP (range = [29:68]%). Similarly, at the 1M time point (N_1m_ = 15), five participants (33.3%) had complete HP (range = (79:125)%), and ten participants (66.7%) had partial HP (range = (31:70)%). At the 3M time point (N_3m_ = 14), five participants (35.7%) had complete HP (range = (75:118)%), and nine participants (64.3%) had partial HP (range = (84:02)%).

### 3.2. Six-Month Follow-Up Data

**Figure 2:**
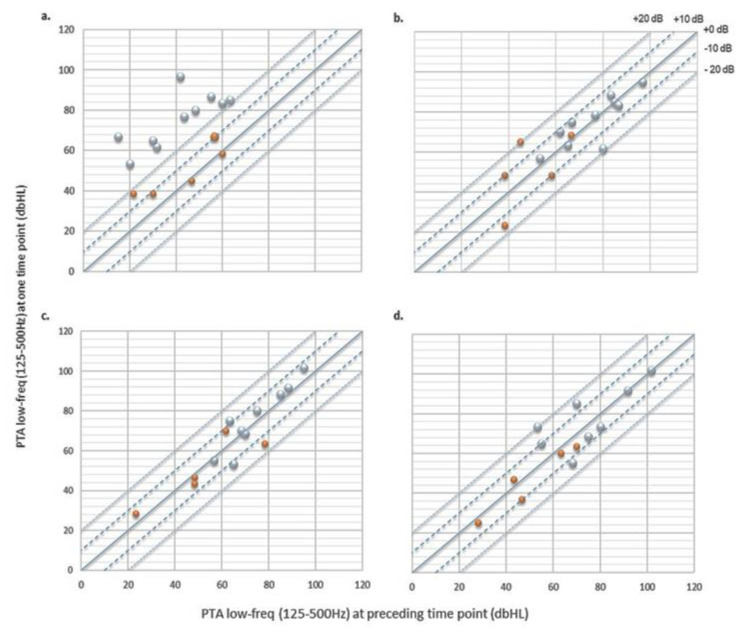
Scatterplots of low-frequency (125–500 Hz) pure-tone audiometry (PTA) at one time point vs. preceding time point: (**a**) activation vs. pre-operative thresholds (*n* = 15); (**b**) 1M after activation vs. activation thresholds (*n* = 15); (**c**) 3M vs. 1M after activation thresholds (*n* = 14); (**d**) 6M vs. 3M after activation thresholds (*n* = 13). The solid line indicates no change in PTA, the dashed lines refer to 10 dB shift in PTA and the dotted lines to 20 dB shift in PTA. Orange circles indicate participants with complete hearing preservation and grey circles participants with partial hearing preservation as defined in the HEARRING group [18].

At the 6M time point (N_6m_ = 13), all participants had measurable hearing thresholds. Figure 2 describes their hearing thresholds from pre-operative visit to the 6M time point. Linear mixed-effect analysis showed that changes in hearing thresholds between visits were not all similar with at least one change in the four visits as statistically significantly different from others (F (4, 408) = 26.6, *p* < 0.001) and were as well not all similar with at least one change in the five testing frequencies as statistically significantly different from others (F (6, 408) = 257.1, *p* < 0.001) with a significant interaction effect between frequency and visit (F (24, 408) = 2.3, *p* = 0.001). Post hoc pairwise multi-comparisons indicated that changes at activation visit, at 1M, at 3M and at 6M time points, were significantly higher from hearing thresholds measured at the preoperative visit between 125 Hz and 1000 Hz. No significant differences were found at both 2000 Hz and 4000 Hz along the follow-up. No significant difference was found between post-operative hearing thresholds. 

At 6M time point (N_6m_ = 13), five participants (38.5%) had complete HP (range = (75:118)%), and eight participants (61.5%) had partial HP (range = (27:61)%). No participant had total loss of hearing or minimal HP according the HEARRING classification.

**Figure 3:**
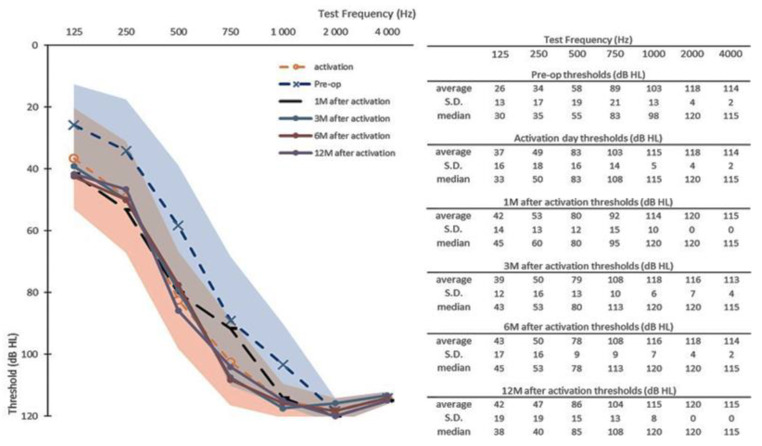
Pre-operative hearing thresholds (Pre-op, *n* = 6), post-operative hearing thresholds at activation day (*n* = 6), at one month (1M) after activation (*n* = 6), at three months (3M) after activation (*n* = 6), at six months (6M) after activation (*n* = 6) and at 12 months (12M) after activation (*n* = 6). Shaded areas represent the maximal and minimal standard deviations of the pre-operative data (in blue) and of the activation day data (in orange). S.D. = standard deviation.

Average HL in regards to the preceding time point within the period of activation visit and the 6M visit was stable (Table 3), as there was no significant change across time points (*t*-tests; HL at 1M vs. at 3M time points (*p* = 0.16), HL at 1M vs. 6M time points (*p* = 0.33) and HL at 3M vs. 6M time points (*p* = 0.18)). Similarly, within the same period, mean HL in the low frequency region (125–500 Hz) was found to be stable (Table 3) with no significant change in mean HL across time points (*t*-tests; HL at 1M vs. 3M time points (*p* = 0.48), HL at 1M vs. 6M time points (*p* = 0.44) and HL at 3M vs. 6M time points (*p* = 0.45)). Figure 2 illustrates hearing threshold shifts within the low-frequency region (125–500 Hz) at one time point vs. the preceding time point. At one month after activation visit (Figure 3), ten participants had PTA increases above +20 dB, and five participants had hearing shifts that were relatively stable between 0 and 20 dB. The latter group consisted of subjects with complete HP as defined by the HEARRING group. All following time points presented equivalent hearing threshold shifts between +20 and −20 dB, with a general tendency of more subjects within the (0, −20 dB) range at the 6M time point.

### 3.3. Twelve-Month Follow-Up Data

At the 12M time point (N_12m_ = 6), all remaining participants had measurable hearing thresholds, as illustrated in Figure 3. Overall, their average HL over 12 months’ follow-up in comparison with the preceding time point was relatively stable (Table 3), both in full range PTAs and PTAs of low-frequency (125–500 Hz). No participant had total loss of hearing or minimal HP according the HEARRING classification, three participants had complete HP (range = (78:111)%) and three participants had partial HP (range = (42:55)%).

### 3.4. Functional Hearing and Low-Frequency Hearing Preservation

**Figure 4:**
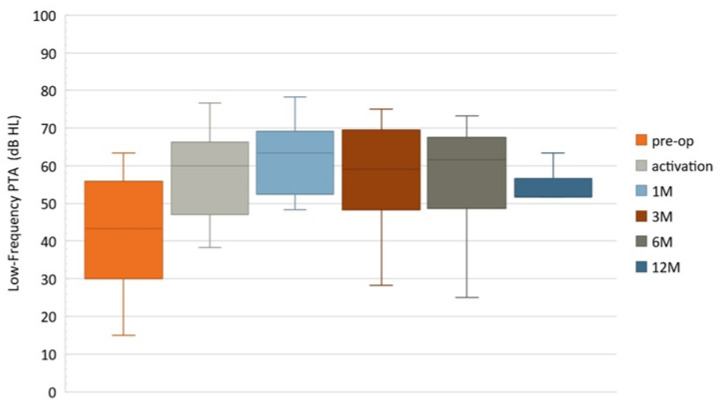
Boxplots of low-frequency (125–500 Hz) pure tone audiometry for participants with functional hearing ≤ 80 dB (HL).

Figure 4 describes all PTAs of low-frequency (125–500 Hz) for participants with functional HP ≤ 80 dB (HL). At the pre-operative time point, all fifteen participants had functional HP with mean PTA low-frequency (125–500 Hz) = 42 dB HL (SD ± 16, median = 43). A total of 66.7% of participants presented functional HP (mean= 57 dB HL (SD ± 13, median = 60)) at activation time point (Nact = 15) and 73.3% participants at the 1M time point (N1m = 15; mean = 60 dB HL (SD ± 16, median = 63)). Note that one participant (Subject 4, Table 2) had nonfunctional HP at activation but presented functional hearing one month later (Subject 4, PTA low-frequency (125–500 Hz) = 80 dB HL at activation, PTA low-frequency (125–500 Hz) = 62 dB HL at the 1M time point). Subject 4 had preserved functional HP over the rest of the follow-up. A total of 71.4% of participants had functional HP at the 3M time point (N_3m_ = 14 (mean = 57 dB HL, SD ± 15, median = 59)) and 76.9% participants at the 6M time point (N_6m_ = 13 (mean = 57 dB HL, SD ± 16, median = 62)). A total of 83.3% of participants had low frequency PTAs ≤ 80 dB (HL) at the 12M time point (N1_2m_ = 6, mean = 53 dB HL (SD ± 9, median = 52). For all subjects with functional hearing, standard HP evaluations showed that at activation (N_act_ = 10), around 60% of subjects had at least partial HP (0–30 dB) and approximatively 40% minimal HP (>30 dB). At the 1M time point (N_1m_ = 11), around 55% of subjects had at least partial HP and 45% minimal HP. At the 3M time point (N_3m_ = 11), 64% of subjects had at least partial HP and 36% minimal HP. At the 6M time point (N_6m_ = 10), around 70% of subjects had at least partial HP and 30% minimal HP. At the 12M time point (N_12m_ = 5), around 80% of subjects had at least partial HP and 20% minimal HP.

## 4. Discussion

To the best of our knowledge, this is the first study that investigated HP in unilateral adult CI recipients with only the EVO™ electrode array up to 12-months follow-up. The EVO^TM^ electrode array was originally designed for less traumatic insertions [16] and satisfying HP scores [17]. Combining soft surgery and appropriate electrode arrays, among other recent recommendations like the use of the dexamethasone [17,22,23], are key to increasing HP probability after implantation and on a long term. One key measure being hearing thresholds, it is noteworthy that both low-frequency range and full-frequency range including higher frequencies should both be tested. If the first range usually represents the functional hearing abilities of a subject, the second considers the full spectrum that is necessary in the daily environment and highlights cochlear integrity [24].

### 4.1. Hearing Preservation Stability over Follow-Up

With full range PTAs, none of the study participants had complete loss of hearing or minimal HP at any time point during follow-up with an overall 100% HP (both partial and complete) detection following the HEARRING group classification. Despite the different number of tested participants, the distribution of HP scores along the different time points was reasonably equivalent. Although the current sample size is quite limited, these data are mostly aligned with the evidence reported in the literature on comparable electrode lengths and CI procedures [25,26,27]. 

Complete low-frequency HP was obtained in (33–40%) range at the early follow-up, in 38.5% at 6M and 66.7% at 12M. Higher rates of complete low-frequency HP at 1 month (68%) have been reported in a retrospective study but with different inclusion criteria (PTA 125–750 Hz) and several types of electrode arrays [28]. Another study using a slim mediolar electrode (*n* = 17), reported 41% of complete low-frequency HP at 43 days (range 3–93) and at 582 days (range 229–1041) after implantation. Using different classification techniques, other studies reported comparable complete HP rates ranging from 25 to 77% [9,17,21,29].

At activation, the HL shift in this study was 11 dB for full range PTA and 25 dB for PTAs of low-frequency (125–500 Hz). These shifts remained stable up to 6M after activation with no significant deterioration. This highlights the fact that the major hearing deterioration could occur in the early months after surgery. Interestingly, the subjects who chose to remain with the EAS up to 12M after activation presented less hearing deterioration with lower shifts for both full range PTA (mean: 7.4 dB) and PTAs of low-frequency (125–500 Hz) (mean: 16.7 dB). Other studies presented comparable HL values [19,28,30].

It is noteworthy to point to the difference of PTA criteria on inclusion across studies and its impact on establishing HP profiles of similar electrode arrays on a larger scale. Differences in the choice of assessment of full range PTA or low-frequency PTA at inclusion, and which frequency range, constitute a major obstacle to perform meta-analysis and draw a larger picture.

### 4.2. Functional Hearing Preservation

One of the limits of the HEARRING classification is that it does not indicate functional hearing. For instance, Subject 14 had partial HP from activation to the 6M time point and yet presented no functional hearing with PTAs of low-frequency (125–500 Hz) ≥80 dB HL. This could explain why this participant chose not to carry on with the EAS, as we could assume there was no amplification benefit. The main challenge remains on how to define functional hearing. There are several recent recommendations in the literature on choosing one definition over the other: median in low frequency hearing at 250 and 500 Hz of 70 dB or better [31], or pure-tone threshold no poorer than 85 dB HL at 250 Hz [24], or PTAs of low-frequency (125–500 Hz) ≥80 dB HL [19], among others (e.g., [32]). The definition is rather arbitrary, may change the outcomes of a dataset from one formula to another and complicate literature comparisons. Determining a common formula for functional hearing seems to be crucial, especially given that this variable can be very informative to the success of EAS use and the atraumatic nature of an electrode array. Considering the EAS eligibility criterion for the current study (PTAs of low-frequency (125–500 Hz) ≤ 70 dB at inclusion), the formula used by Iso-Mustajärvi et al. [19] seemed most interesting for the current dataset, as all hearing thresholds ≤500 Hz could be included. A total of 66.7%, 73.%, 71.4%, 76.9% and 83.3% of the participants presented functional hearing at activation at 1M, 3M, 6M and 12M time points, respectively. Interestingly, the detection of functional hearing increased over time in the different sample sizes who chose not to quit the study and keep the EAS. Iso-Mustajärvi et al. [19] report that 83% of tested ears with a slim mediolar electrode had functional hearing at activation and 82% at latest follow-up (mean 582 days). Considering the reported wide range of the activation day (range 3–93) and of their follow-up time point (range 229–1041), the current data are comparable with theirs. On a larger sample size, Helbig et al. [9] found that functional low-frequency residual hearing (PTAs low-frequency (125–500 Hz) < 80.0 dB HL) was 85.3% postoperatively, 87.9% after the 12M time point and 95.0% for longer follow-up. Lee et al. [25] assessed HP on 34 ears and found that 41% had functional hearing (no poorer than 85 dB HL at 250 Hz), while 59% did not at one-year post-activation. Looking to the current dataset at 12M, all six participants had better hearing thresholds than 85 dB HL at 250 Hz. Moran et al. [31] reported that 39.5% of 86 tested participants presented functional hearing as median hearing at 250 and 500 Hz of 70 dB or better at 3 months postoperatively. Using the same method, Harrison et al. [26] found 32.7% of their participants with functional hearing postoperatively. In the current dataset, 35.7% of the participants presented functional hearing using the above definition at 3M. In sum, the current study presents comparable evidence to the literature when comparing different methods of functional preservation.

All subjects with functional hearing presented at least partial HP or minimal HP and no case of loss of residual hearing. Although the sample size is comparatively smaller in the current study, these outcomes are relatively aligned with recent studies reporting on hearing preservation and using the same method [9,21,28,30]. 

### 4.3. Correlation with Age

When investigating correlations between potential predictive factors on hearing outcomes, only one significant correlation was found between age at inclusion and PTAs of low-frequency (125–500 Hz) at activation (r = 0.56; *p* = 0.03). Iso-Mustajärvi et al. [19] also found a moderate correlation between age and residual hearing but at the end of the follow-up. However, the predictive impact of age on hearing preservation remains uncertain, as several studies have shown better outcomes on residual hearing [20,33], while other studies did not report any significant impact [26,34,35]. Although the literature reported other predictive factors such as duration of hearing loss [36,37], they were not highlighted in the current dataset, possibly due the limited number of participants. 

### 4.4. EAS Adherence

In the current study, subjects could quit the protocol at any time of the follow-up and were giving the choice between keeping the EAS or being fitted with an ES starting from 6M. At all the time points, all participants were eligible for EAS (i.e., eligibility criterion being at least one detectable hearing threshold). At the 6M time point, 92.9% (13/14) of participants decided to keep the EAS when given the choice, out of which 46.2% kept it at 12M. Spitzer et al. [38] reported EAS eligibility (postoperative thresholds ≤75 dB HL for both 125 and 250 Hz) in 64.5% (49/76) at activation, 55.7% (44/79) at 3 months postoperatively and 51% (24/47) at 12 months postoperatively. At each time point, the authors reported that approximately half of the participants chose to use EAS with approx. 20% at 12 months postoperatively. A total of 78% of CI ears in Iso-Mustajärvi et al. were EAS eligible, of which 72% were fitted with an EAS and only 44% chose to keep the EAS. Harrison et al. [26] reported that only 2 of 17 eligible subjects chose to use EAS. Out of 32 EAS users in the study by Roland et al. [32], 84% were still using the EAS after 12-month follow-up and 72% after 5 years. Mamelle et al. [28] reported that 20/44 of their participants stopped using EAS, 6 of which were because of aesthetic issues, 8 of which were because of acoustic discomfort and 6 of which were because of total HL. Regardless of the several EAS eligibility criteria, the concept of adherence to EAS use has been briefly discussed in the literature, and limited evidence has been reported on EAS users’ reasons to drop. In the current study, two reasons mostly underlined the choice of our participants in quitting the study. The first one, and in most cases, was personal constraints. The second one was hoping for a better benefit with ES use whether because of their belief that no benefit is coming from EAS or because they believe the benefit will be better with ES. Spitzer et al. [38] investigated the reasons behind the rejection of EAS use. A total of 29% of rejections was because of hearing issues (21% borderline thresholds; 8% of fluctuating hearing), and 46% were because of discomfort or equipment preference. Regardless of the EAS brand or the electrode type, these figures highlight the urgent need to focus on whether to improve equipment or EAS criteria to benefit more EAS eligible candidates. Interestingly, the current study did not define strict EAS criteria after activation, yet a comparable number of EAS adherents was found with several studies with stricter criteria. 

## 5. Conclusions

The EVO^TM^ electrode array is designed for hearing preservation surgeries, and the present data show that all participants had measurable hearing levels with complete and partial preservation profiles. Thresholds remained stable over the follow-up period, with a steady hearing threshold shift suggesting that hearing loss was stable along the follow-up period. Participants who chose to remain with EAS had better hearing preservation profiles and lower hearing threshold shift. This study confirms the atraumatic profile of the EVO^TM^ electrode array, but other studies on larger populations should be performed.

## Figures and Tables

**Table 1:** Demographic characterization of all included participants. (*n* = 15).

ID.	Age	Gender	Age at Deafness Onset (Years)		Etiology	HL Evolution Severe/Profound	
			R	L		R	L
1	76	M	66	1	Presbyacousis	progressive	progressive
2	55	F	37	37	Genetic	sudden	sudden
3	53	M	1	1	Unknown	progressive	progressive
4	78	F	61	66	Unknown	n.a	n.a
5	39	F	4	4	Meningitis	progressive	progressive
6	69	F	42	42	Unknown	progressive	progressive
7	34	M	5	5	Unknown	sudden	progressive
8	71	F	30	1	Unknown	progressive	sudden
9	86	F	47	47	Unknown	sudden	sudden
10	36	F	n.a	n.a	n.a	n.a	n.a
11	61	M	34	34	Unknown	progressive	progressive
12	46	F	1	1	Ototoxic	progressive	progressive
13	57	M	45	45	Genetic	sudden	sudden
14	81	M	69	69	Unknown	progressive	progressive
15	72	F	53	53	Unknown	progressive	progressive

M: Male; F: Female; R:Right; L:Left; HL: Hearing loss.

**Table 2:** Individual PTAs for all included participants. (*n* = 15). Pre-op = pre-operative visit. Act = activation day visit. 1M, 3M, 6M and 12M refer, respectively, to one-month, three-month, six-month and twelve-month visits after activation. x refers to participants out of study. PTA = pure-tone audiometry; HL = hearing loss.

ID	CI Ear	Low-Frequency PTA [125–500 Hz]						Full Range PTA (dB HL) [125–4000 Hz]					
		Pre-op	Act	1M	3M	6M	12M	Pre-op	Act	1M	3M	6M	12M
1	right	56,7	66.7	68.3	70.0	85.0	x	77.7	83	83.4	88.6	90.5	x
2	left	46.7	45.0	65.0	53.3	73.3	85.0	78.2	79.1	85	88	93.4	96.6
3	left	43.3	76.7	78.3	63.3	60.0	x	81.1	90.9	93.6	88.4	85.9	x
4	left	48.3	80.0	61.7	70.0	63.3	63.3	79.1	93	88.9	84.5	86.8	85.9
5	left	60.0	58.3	48.3	43.3	46.7	51.7	89.8	89.2	84.8	85.2	86.1	87.5
6	left	15.0	66.7	75.0	80.0	73.3	x	63	80.2	81.1	93.9	83.2	x
7	right	30.0	38.3	48.3	46.7	36.7	40.0	81.6	83.9	84.8	84.3	82	82.5
8	right	63.3	85.0	85.0	88.3	x	x	60.2	83.6	84.5	85.7	x	x
9	left	55.0	86.7	83.3	x	x	x	88.4	97	96.1	x	x	x
10	left	31.7	61.7	70.0	68.3	55.0	51.7	74.5	89.8	92	91.6	88.4	84.1
11	right	21.7	38.3	23.3	28.3	25.0	x	65.9	71.4	68.6	66.8	66.6	x
12	right	20.0	53.3	56.7	55.0	65.0	56.7	71.6	83.9	83	86.6	86.8	88.9
13	left	41.7	96.7	95.0	101.7	101.7	x	75.2	99.8	99.3	101.1	100.7	x
14	left	60.0	83.3	88.3	91.7	91.7	x	69.5	88	90.5	91.6	97.5	x
15	left	30.0	65.0	63.3	75.0	68.3	x	70.7	88.2	88.4	93.9	92	x

**Table 3:** Average hearing loss over follow-up vs. preceding visit (dB HL) up to 6 months follow-up (*n* = 13) in both full range and low-frequency pure-tone audiometry.

Time Point:	Activation	1M	3M	6M
Full range Hearing loss (25–4000 Hz) (dB)				
average	11	0.2	1.6	−0.4
S.D.	7.8	2.9	4.7	4.2
Low-Frequency Hearing loss (125–500 Hz) (dB)				
average	25	0.9	0.4	−0.1
S.D.	18.1	10.6	7.7	9.9

## Data Availability

All data generated or analysed during this study are included in this article and its Supplementary material files. Further enquiries can be directed to the corresponding author.

## Supplementary Materials

The following are available online at https://www.mdpi.com/article/10.3390/jcm10235604/s1. Table S1. Hearing preservation scores and classification according to the HEARING group classification [18] over the 12 months’ follow-up at full range hearing loss (125–4000 Hz) and low-frequency PTA (125–500 Hz).
