# Supplementary material for: One Year Assessment of the Hearing Preservation Potential of the EVO Electrode Array
Source: J Clin Med. 2021 Nov 29;10(23):5604. doi: 10.3390/jcm10235604 (PMC8658238; doi:10.3390/jcm10235604)
Supplement: Supplementary file 1 [file jcm-10-05604-s001.zip › jcm-1438986-supplementary.pdf]

Supplementary materials

Table S1. Hearing preservation scores and classification according to the HEARING group classification ((Skarzynski et al., 2013) over the 12 months' follow-up at full range hearing loss (125-4000 Hz) and low-frequency PTA (125-500 Hz).

ID	Hearing Preservation (HP %) at full range PTA					class				
	activation	M1	M3	M6	M12	activation	M1	M3	M6	M12
1	84	82	66	61	x	complete	complete	partial	partial	x
2	97	79	69	52	42	complete	complete	partial	partial	partial
3	66	57	75	84	x	partial	partial	complete	complete	x
4	55	68	82	75	78	partial	partial	complete	complete	complete
5	102	125	123	118	111	complete	complete	complete	complete	complete
6	63	61	34	57	x	partial	partial	partial	partial	x
7	92	89	90	98	97	complete	complete	complete	complete	complete
8	53	51	49	x	x	partial	partial	partial	x	x
9	60	64	x	x	x	partial	partial	x	x	x
10	57	51	52	61	73	partial	partial	partial	partial	partial
11	88	94	98	99	x	complete	complete	complete	complete	x
12	68	70	61	60	55	partial	partial	partial	partial	partial
13	29	31	26	27	x	partial	partial	partial	partial	x
14	55	48	46	31	x	partial	partial	partial	partial	x
15	56	55	41	46	x	partial	partial	partial	partial	x

ID	Hearing Preservation (HP %) at low-freq PTA					class				
	activation	M1	M3	M6	M12	activation	M1	M3	M6	M12
1	84	82	79	55	x	compelete	complete	complete	partial	x
2	102	75	91	64	48	compelete	complete	complete	partial	partial
3	57	54	74	78	x	partial	partial	partial	complete	x
4	56	81	70	79	79	partial	complete	partial	complete	complete
5	103	119	128	122	114	compelete	complete	complete	complete	complete
6	51	43	38	44	xx	partial	partial	partial	partial	x
7	91	80	81	93	89	compelete	complete	complete	complete	complete
8	62	62	56	x	x	partial	partial	partial	x	x
9	51	56	x	x	x	partial	partial	x	x	x
10	66	57	58	74	77	partial	partial	partial	partial	complete
11	83	98	93	97	x	compelete	complete	complete	complete	x
12	67	63	65	55	63	partial	partial	partial	partial	partial

**13**	30	32	23	23	x	partial	partial	minimal	minimal	x
**14**	61	53	47	47	x	partial	partial	partial	partial	x
**15**	61	63	50	57	x	partial	partial	partial	partial	x
